# The geological factors affecting gas content and permeability of coal seam and reservoir characteristics in Wenjiaba block, Guizhou province

**DOI:** 10.1038/s41598-023-46470-9

**Published:** 2023-11-03

**Authors:** Cong Feng, Xijian Li, Rui Yang, Junjie Cai, Hao Sui, Honggao Xie, Ziyi Wang

**Affiliations:** 1 College of Mining, Guizhou University, Guiyang, 550025 China; 2 College of Resource and Environmental Engineering, Guizhou University, Guiyang, 550025 China; 3 Guizhou Engineering Center for Safe Mining Technology, Guiyang, 550025 China; 4 Guizhou Water Mine Aorian Clean Energy Limited Company, Guiyang, 550025 China

**Keywords:** Environmental sciences, Environmental social sciences

## Abstract

The gas content and permeability of coal reservoirs are the main factors affecting the productivity of coalbed methane. To explore the law of gas content and permeability of coal reservoirs in the Zhijin area of Guizhou, taking No.16, No.27 and No.30 coal seams in Wenjiaba mining area of Guizhou as the engineering background, based on the relevant data of coalbed methane exploration in Wenjiaba block, the geological structure, coal seam thickness, coal quality characteristics,coal seam gas content and permeability of the area were studied utilizing geological exploration, analysis of coal components and methane adsorption test. The results show that the average thickness of coal seams in this area is between 1.32 and 1.85 m; the average buried depth of the coal seam is in the range of 301.3–384.2 m; the gas content of No.16 and No.27 coal seams is higher in the syncline core. The gas content of the No.30 coal seam forms a gas-rich center in the south of the mining area. The buried depth and gas content of coal seams in the study area show a strong positive correlation. Under the same pressure conditions, the adsorption capacity of dry ash-free basis is significantly higher than that of air-dried coal. The permeability decreases exponentially with the horizontal maximum principal stress and the horizontal minimum principal stress. The horizontal maximum primary stress and the flat minimum prominent stress increase with the increase of the buried depth of the coal seam. The permeability and coal seam burial depth decrease exponentially. This work can provide engineering reference and theoretical support for selecting high-yield target areas for CBM enrichment in the block.

## Introduction

Coalbed methane is an important clean energy. The geological resources of coalbed methane in China reach 36.8 trillion m^3^, of which the coalbed methane resources in coal mining areas exceed 16 trillion m^3^, accounting for 43.5% of the total coalbed methane resources in China^1,2^. To ensure the safe production of coal mines and ensure the energy demand of China’s rapid economic development, it is of great significance to increase the growth of coalbed methane extraction technology and increase the production of coalbed methane in coal mining areas to accelerate the healthy development of China’s ecological civilization and promote the process of achieving the goals of “carbon peak” and “carbon neutralization”^3–6^.

Guizhou is known as the “Jiangnan Coal Sea”. The coal seam is rich in coalbed methane resources, reaching 3.15 trillion cubic meters, accounting for about 10% of the country’s coalbed methane geological resources, ranking third in the country. It is the reserve area of China’s future key development of coalbed methane industrialization base^7,8^. The daily production of coalbed methane wells in southwestern Guizhou is close to 5000 m^3^, and it is also close to 1000 m^3^ in northwestern Guizhou^9^. In the process of coalbed methane development, gas content not only determines the reserves of coalbed methane, but also is an important geological parameter affecting the production of coalbed methane and the main index for evaluating coal seams. At the same time, it also directly determines whether coalbed methane can be effectively developed^10^. Its determination method is mainly divided into two categories, namely, direct method and indirect method. The direct method is to measure the gas content in coal samples after desorption^11–13^. The indirect method is to predict the gas content of coal seam by gas emission and adsorption isotherm curve^14–16^. Permeability is a key factor to determine the development process and ultimate recovery of coalbed methane. It is often used to characterize the basic permeability of coal reservoirs by the level of permeability^17,18^. There are two main methods to evaluate the gas content of coal seam based on logging data analysis: one is regression analysis method. Specifically, Lei et al.^19^ used laboratory measurement method to measure the basic gas parameters and coal quality indexes of 24 coal samples from coal mines in Hancheng area of Shanxi Province, and used SPSS software to use stepwise multiple linear regression method for statistical analysis. At the same time, a mathematical model for rapid prediction of coal seam gas content was established. Zhang et al.^20^ established a calculation model of gas content in deep coal seam with correction coefficient based on the relationship between measured gas saturation and buried depth of coal seam by nonlinear analysis method. Meng et al.^21^, Akdas et al.^22^, Wei et al.^23^ used artificial intelligence algorithms, such as support vector regression, machine learning, neural network of PCA-AHPSO-SVR and other methods to predict coal seam gas content, and achieved good application results in some areas at home and abroad. The second is the isothermal adsorption method. Specifically, Zhang et al.^24^ established an analysis model based on Langmuir adsorption theory and a numerical method to characterize the staged desorption of coalbed methane based on the equivalent desorption rate curve according to the results of isothermal adsorption experiments. Based on the isothermal adsorption theory, Xu et al.^25^ quantitatively divided the reasonable drainage stage and desorption stage, developed a systematic drainage system, and achieved good drainage effect in engineering practice. Feng^26^ calculated the critical desorption pressure by combining the coalbed methane production model and the isothermal adsorption model, and accurately characterized the adsorption characteristics of coalbed methane by Langmuir equation. Zhao et al.^27^ carried out quantitative characterization of full pore size distribution and isothermal adsorption experiments, corrected the calculation of gas content in deep coal, and discussed the difference of gas content calculated by different methods and its pore size effect.

In summary, many researchers have used laboratory tests, field tests, and other methods to study the geological conditions of the reservoir and have achieved rich research results. This paper aims to deeply analyze the main geological factors affecting the gas content and permeability of coal reservoirs in the Wenjiaba area. Based on the previous contributions and combined with the geological situation of the mining area, the reservoir geological conditions of No.16, No.27 and No.30 coal seams in this area are analyzed, and the geological exploration, analysis of coal components, and methane adsorption test methods are used to comprehensively and comprehensively study the gas content and permeability of coal seams. The influence of geological factors on the gas content and permeability of coal seams in the Wenjiaba area is discussed in order to provide a reference for similar geological projects.

## Geological survey of mining area

**Figure 1:**
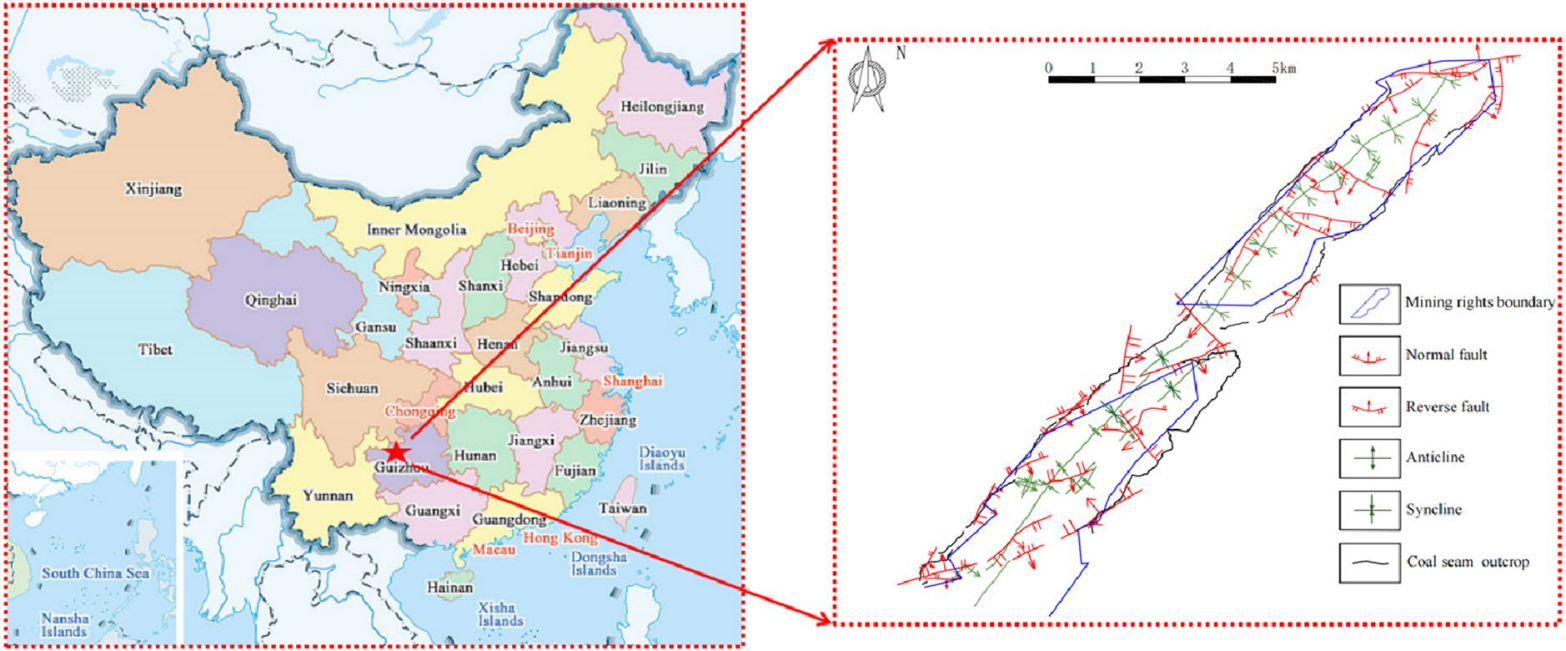
Location and regional structure of mining area.

Guizhou is one of the largest coal resource provinces in southern China and one of China's coal resource and coalbed methane enrichment areas. The mining area of Guizhou Province is representative^28,29^. The study area is located in the east wing of Qianxi Mountain, which is roughly extended by more than 50 km in the direction of 40° northeast. In the geotectonic unit, it belongs to the south margin of the Yangtze block (grade I), Qianbei uplift (grade II), Zunyi fault arch (grade III) and Bijie northeast tectonic deformation zone (grade IV). Under the influence of the primary pressure in the northwest-southeast direction, a series of Cathaysian anticlines and synclines with axes roughly parallel to each other and distributed in the northeast 45° order are formed in the mining area. The northwest wing is steep, the structure is complex, the tensile and torsional faults coexist, and most are strike faults destructive to the coal seam. The southeastern wing is gentle, the structure is simple, generally dominated by tensile faults, and the fault distance is small, primary oblique faults^30^. From west to east, there are 20 folds, such as the Zhangwei anticline, Santang syncline, Houzhai anticline and so on. When the fold axis is in the northeast direction, it is a gentle open fold, and the scale of the nearly east–west compressional fault is the largest, generally a high-angle thrust fault. When the fold axis is northeast-northeast east, the syncline fold is open, the anticline is tight, and the fracture direction is primary consistent with the fold axis^31^. The location and regional structure of the mining area are shown in Fig. 1.

**Figure 2:**
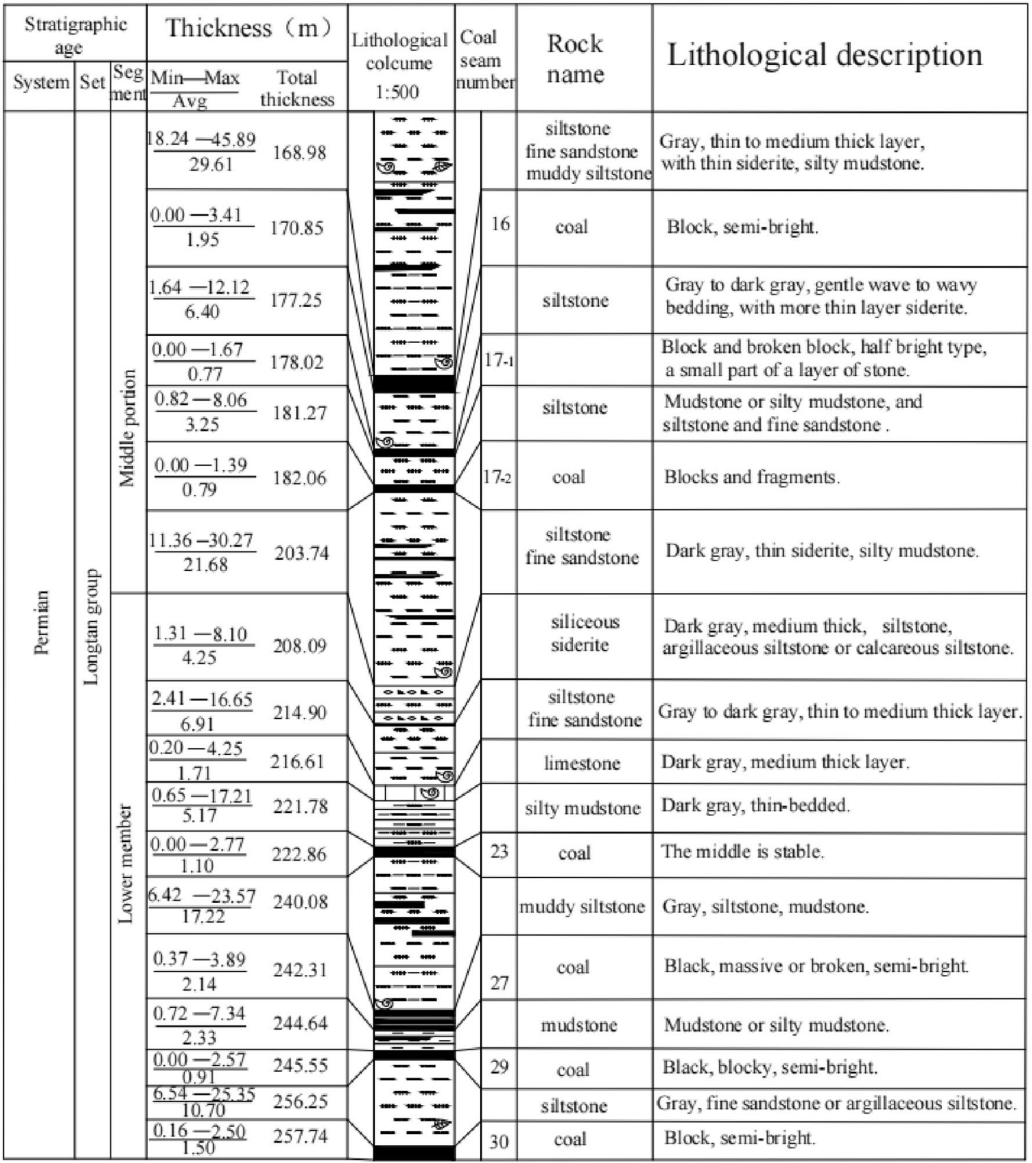
Composite formation histogram.

As shown in Fig. 2, the mining area is dominated by Permian^32^. From bottom to top are the Maokou Formation of Middle Permian, Emeishan Basalt Formation, Longtan Formation and Changxing Formation of Upper Permian. The coal seams in the study area are mainly in the middle and lower sections of the Longtan Formation of the Upper Permian. The Longtan Formation is the primary coal-bearing stratum exposed on both sides of the syncline. The thickness is generally 246–314 m, of which 6 layers can be mined. The lithology is composed of fine sandstone, siltstone, silty mudstone, argillaceous siltstone, mudstone, carbonaceous mudstone, coal seam, bauxite mudstone, bauxite rock, limestone, flint limestone, marl and siliceous siderite.

## Results analysis

### Coal quality characteristics

The coal seams of each target layer in the mining area are black and gray-black, and their morphology is mainly blocky, with a small amount of fragments and powder. The coal seam structure of each target layer is mostly medium-fine banded structure, and a small amount is fine banded structure; glass luster is the main, followed by metal-like brilliance; the fracture of the W1 thriving area is staggered chiefly, and the W2 healthy area is mostly shell-like; the endogenous and exogenous fractures in the W1 healthy area are more developed, and the local filling is thin film, mesh and vein calcite; pyrite generally occurs in spherical, lenticular, nodular, nodular, layered, and fine-grained forms. The coal rock composition is dominated by bright and dark coal, followed by specular coal and silk carbon. The macroscopic coal rock types of the W1 thriving area's target layer are mainly semi-bright and semi-dark-semi-bright coal. The W2 thriving area is dominated by bright-semi-bright coal.

### Water quality characteristics and roof lithology distribution

The research block is located in the Longtan Formation of the Permian system. Near the axis of the syncline, the water-richness of limestone and ferruginous siliceous rocks such as Biaosanxia, Biaosanxia and Biaosi is strong, and the moisture content of other sand and mudstone is relatively weak, which is a vulnerable aquifer. The group is mainly clastic rock, the shallow part is dominated by weathered fissure water, and the deep part is dominated by tectonic fissure water. The lithology of this group changes significantly. Under the influence of the sedimentary thickness of the sandstone body and the multi-cycle of transgression and regression, the thickness of the aquifer is unstable and mainly occurs as a convex mirror. The moisture content will be relatively large in the areas affected by structural faults and stress failure. When the deposit is mined in these areas, the mine water output will increase more than expected. In addition, the upper, middle and lower sections of the Longtan Formation contain several layers of limestone and flint limestone with different thicknesses, especially the upper area of limestone. The limestone develops several karst caves and karst holes, one of the crucial direct water filling sources for coal mining.

The roof and floor rock groups of No.16, 27, and 30 coal seams comprise fine sandstone, siltstone, argillaceous siltstone and silty mudstone. Among the coal seam roof and floor lithologies, fine sandstone and siltstone belong to semi-hard rock mass with good stability. Still, argillaceous siltstone and silty mudstone belong to weak rock group, easily weathered, broken, expanded or disintegrated in water, and have poor stability. Among them, the argillaceous limestone of the No.30 coal seam is a hard rock group with good stability. Generally, the strength of the roof and floor of No.16, 27, and 30 coal seams is moderate.

The lithology of coal-bearing strata in the study area has a medium deviation in mechanical strength and stability deviation. The overlying strata are complicated and have high mechanical strength. The rock mass in the area is mainly a layered structure, and the force changes significantly. Its stability depends on the weak surface between layers, the tectonic belt and weathering degree of rock mass. The roof and floor of the coal seam are mainly rock mass with medium mechanical strength; the faults in the area are weakly developed, and the joint fissures near the coal seam outcrop are formed. The stability of the slope is general, and the strength of the rock strata of different coal seams in the surrounding rock of the roadway is additional, with hard rock, medium hardness rock, soft rock and other rocks. The engineering geological type of the mining area is the third type of clastic rock, the primary layered rock.

### Coal seam thickness

There are 21–44 coal seams in the W1 thriving area, generally about 30 layers. The total coal thickness is 13.32–33.58 m, with an average of 24.30 m, and the coal-bearing coefficient is 8.6%. There are six layers of minable coal seams, divided into two types: the whole area can be mined, and most can be mined. The total thickness of minable coal seams is 7.17–25.93 m, with an average of 16.26 m, and the minable coal content coefficient is 5.75%. Among them, the whole area can be mined as 6, 7, 16, 27, 30 coal seams. The W2 thriving area contains 24–44 layers of coal, generally 30–33 layers, with a total coal thickness of 17.03–34.44 m, an average of 25.74 m, and a coal-bearing coefficient of 5.91–12.24%, generally 8–9%. No.16 and No.27 coal seams are mineable coal seams in the whole area, and No.30 is the most mineable coal seams. The characteristics of coal seams are shown in Table 1.

**Table 1:** Coal seam characteristics.

Well area	Number of coal seam	Minimum thickness/m	Maximum thickness/m	Number of layers of dirt band	Texture of coal seam	Stable degree	Degree of recoverable
W1	16	1.09	2.89	0–2	Plain	Stabilization	District admissible
	27	0.60	2.85	0–4	Relatively complicated	Relatively stable	District admissible
	30	0.72	2.07	0–2	Plain	Stabilization	District admissible
W2	16	0.96	4.05	0–3	Relatively simple	Stabilization	District admissible
	27	0.61	3.29	1–7	Complication	Relatively stable	District admissible
	30	0.60	2.71	0–4	Relatively complicated	Stabilization	Mostly admissible

**Figure 3:**
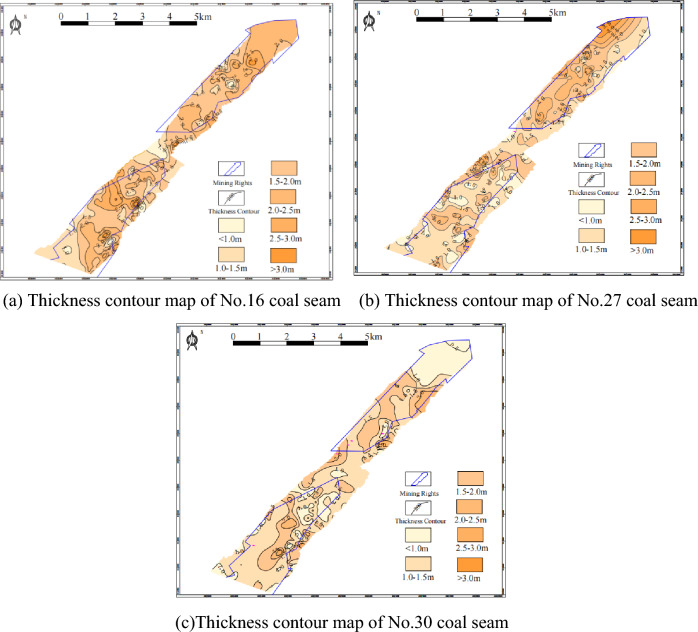
Coal seam thickness contour map of Wenjiaba mining area.

It can be seen from the above table that the No.16 coal seam is located in the middle and lower part of the middle section of the Longtan Formation, and the layer is stable. The thickness is 0.96–4.05 m, with an average of 1.85 m. The structure is simple to relatively simple. The plane shows the coal seam thickens and thins from southwest to northeast. The overall thickness is greater than the other two coal seams. The coal seam thickness contour in this area is shown in Fig. 3a. The No.27 coal seam is located in the middle of the lower section of the Longtan Formation, and the horizon is relatively stable. The thickness of the coal seam is in the range of 0.60–3.29 m, with an average of 1.55 m; generally, there are 1–2 layers of dirt band, and the structure is more complex. On the plane, the coal seam gradually thickens from southwest to northeast. The contour of coal seam thickness in this area is shown in Fig. 3b. No.30 coal seam is located in the middle of the lower section of the Longtan Formation, with a stable horizon. The thickness of the coal seam is 0.60–2.71 m, with an average of 1.32 m. The structure is simple to complex, and the plane shows a trend of thick in the middle and thin around. The contour of coal seam thickness in this area is shown in Fig. 3c.

### Coal seam depth

The burial depth and spatial form of the coal seam refer to the current occurrence state of the coal seam, which is the result of later tectonic movement after the formation of the coal seam^33^. The burial depth of a coal seam affects important reservoir parameters such as coalbed methane content, coal seam permeability, reservoir pressure and reservoir temperature. Still, it also is a crucial parameter in evaluating coalbed methane exploration and development. From the perspective of coalbed methane development, the burial depth of coal seam must meet the requirements of current technical conditions for exploration and development depth.

**Figure 4:**
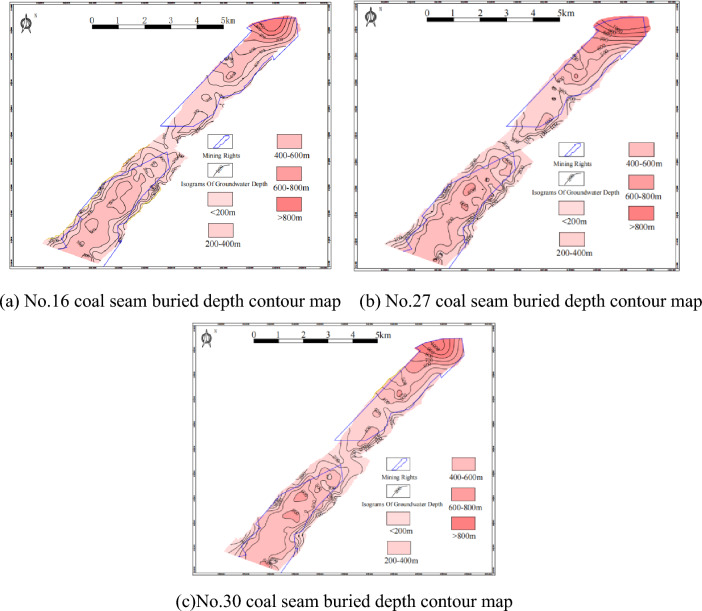
Coal seam buried depth contour map.

The buried depth of the coal seam in Guizhou mountainous area is calculated by the average elevation of the coal seam outcrop minus the peak of the coal seam. The buried depth of coal seam in the study area is mainly distributed in 200–800 m, which is the most favorable buried depth range for commercial development of coalbed methane^34^. From the contour map of the buried depth of No.16, No.27 and No.30 coal seams (Fig. 4), it can be seen that the buried depth of coal seams in the syncline core is more significant, and the wings are shallower. From the statistics of coal and coalbed methane exploration data, it can be seen that the average buried depth of the No.16 coal seam is 301.3 m, the average buried depth of the No.27 coal seam is 366.6 m, and the average buried depth of No.30 coal seam is 384.2 m.

### Coal seam gas content

The overall gas content in the study area is good. According to the experimental results of coal samples as shown in Table 2, the gas content of No.16 coal seam is 12.03–17.52 m^3^/t, with an average of 14.68 m^3^/t; the gas content of No.27 coal seam is 8.12–21.56 m^3^/t, with an average of 15.07 m^3^/t; the gas content of No.30 coal seam is 10.63–21.83 m^3^/t, with an average of 15.40 m^3^/t. The average gas content of No.30 coal seam is higher than that of No.27 and No.16 coal seams, indicating that No.30 coal seam has better development potential than the other two coal seams.

**Table 2:** Statistical table of coal seam gas content in W1 and W2 mines.

Coal seam	Gaseous composition			Gas content ad m^3^/t
	N_2_ (%)	AverageCO_2_ (%)	CH_4_ + C_2_H_6_ (%)	
16	3.55	1.29	84.21–99.87	12.03–17.52
27	4.37	0–8.01	84.16–100	8.12–19.56
30	2.74	0–8.59	87.64–99.85	10.63–19.83

**Figure 5:**
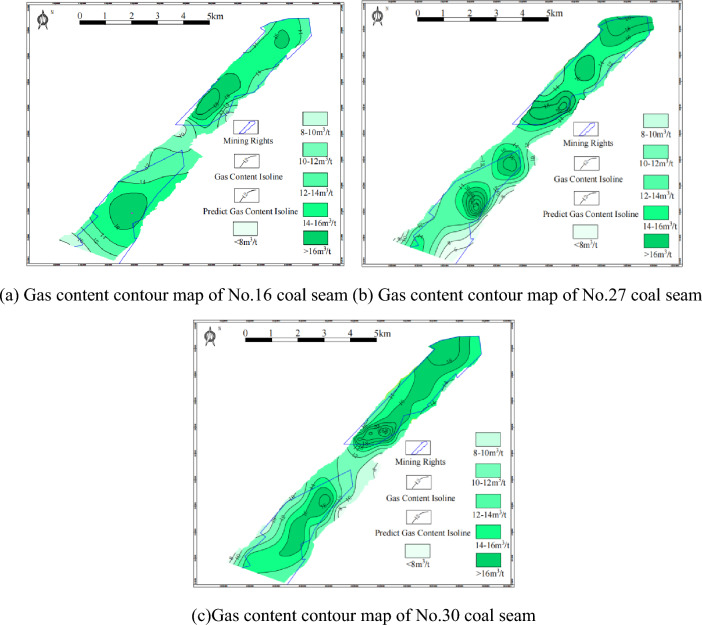
Coal seam gas content contour map.

According to the relevant data, the gas content contour of each coal seam is drawn as shown in Fig. 5. According to Fig. 5a, the gas content of the No.16 coal seam forms a gas-rich center in the south of the W1 mining area. In general, the gas content in the core of the syncline is higher, and the gas content in the W1 mining area is higher than that in the W2 mining area. According to Fig. 5b, the gas content of the No.27 coal seam is higher in the core of the syncline. A gas-rich center is formed in the south of the W1 mining area, and a gas-rich center is created in the middle and north of the W2 mining area. According to Fig. 5c, the gas content of the No.30 coal seam forms a gas-rich center in the south of the mining area.

Combined with Table 2 and Fig. 5, it can be seen that the analysis results of natural desorption gas components of coalbed methane show that coalbed methane is a high-quality coalbed methane resource. The main features are methane, nitrogen, carbon dioxide, heavy hydrocarbons, etc., in which the methane concentration is high, and the rich hydrocarbon content of individual boreholes is also high, the concentration of methane + heavy hydrocarbon can reach 95.93%. Compared with other coal seams, the concentration of methane + heavy hydrocarbons in the No.30 coal seam is higher, with an average of 96.02%. The average CO_2_ concentration of the No.27 coal seam is up to 1.58%, and the average N_2_ concentration is up to 4.37%.

**Figure 6:**
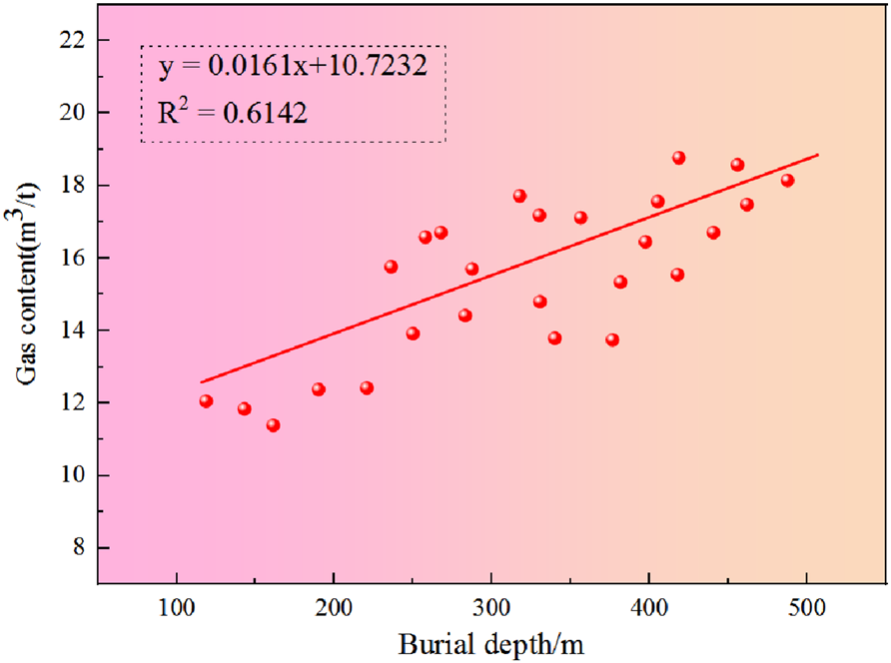
Relationship between buried depth and gas content.

The buried depth of a coal seam has different positive and negative effects on gas adsorption. The reasons may be due to the following two points: (1) Positive effect, that is, with the increase of buried depth, the ground stress also increases, which is conducive to gas adsorption, thereby increasing the gas content of coal seam; (2) Negative effect, that is, with the increase of burial depth, the formation temperature gradually increases. Due to the negative correlation between temperature and adsorption, the amount of adsorbed gas is relatively reduced, and the gas content of the coal seam is reduced. Based on the above considerations, combined with previous studies^35–37^, the strength of the positive effect of ground stress on coal seam gas content is defined as FZ. The negative effect of formation temperature on coal seam gas content is FF, and the critical value of FF = FZ is defined as the required burial depth. When the buried depth is less than the necessary buried depth, the gas content of the coal seam is mainly affected by the buried depth, that is, FZ > FF, and the gas content of the coal seam increases with the increase of the buried depth, which is prone to enrichment. When FZ > FF, the gas content of the coal seam decreases with the addition of buried depth. When FF = FZ, it is the critical buried depth value defined above, and the coal seam gas content reaches the maximum value at this time, and it is also easy to cause enrichment. According to Fig. 6, the buried depth and gas content of coal seams in the W1 thriving area and the W2 thriving area of the study area show a strong positive correlation, with R^2^ = 0.6142. Considering that the value is less than 0.8, the reason may be that coal is a complex macromolecular structure and the microstructure and physical and chemical properties of different coal seams are other, which leads to the dispersion of coal to a certain extent.

### Coal seam adsorption

Domestic and foreign studies have shown that Zhejun et al.^38^ used the extended Langmuir model, the ideal adsorption solution model and the two-dimensional state equation to study the influence of the adsorption model on the reservoir simulation results. Based on methane isothermal adsorption experiments, the supercritical methane adsorption characteristics of medium-rank tectonically deformed coals selected from Huaibei coalfield were analyzed by Lu et al.^39^. Liu et al.^40^ conducted high-pressure isothermal adsorption and desorption experiments at different temperatures for broken coal samples from the Huainan and Huaibei coalfields. Based on the methane adsorption phase density predicted by the intercept method and the theoretical adsorption amount, a mixed model based on different adsorption theories was established.

There is a robust physical adsorption between methane molecules and coal matrix particles. The adsorbed methane in the coal seam constitutes the main body of coalbed methane, which is more than 90%. The isothermal adsorption properties of coal determine the binding strength and dispersion speed of coalbed methane.

When the temperature is constant, the adsorption capacity of coal to methane obeys the Langmuir isotherm equation^41^:

1 $$ V = {{V_{L} \times P} \mathord{\left/ {\vphantom {{V_{L} \times P} {\left( {P_{L} + P} \right)}}} \right. \kern-0pt} {\left( {P_{L} + P} \right)}} $$

In the formula *V* is the theoretical gas content corresponding to the measured reservoir pressure projected onto the adsorption isotherm, m^3^/t; *V*_*L*_ is langmuir volume, m^3^/t; *P*_*L*_ is langmuir pressure, MPa;*P* is Measured reservoir pressure, MPa.

**Figure 7:**
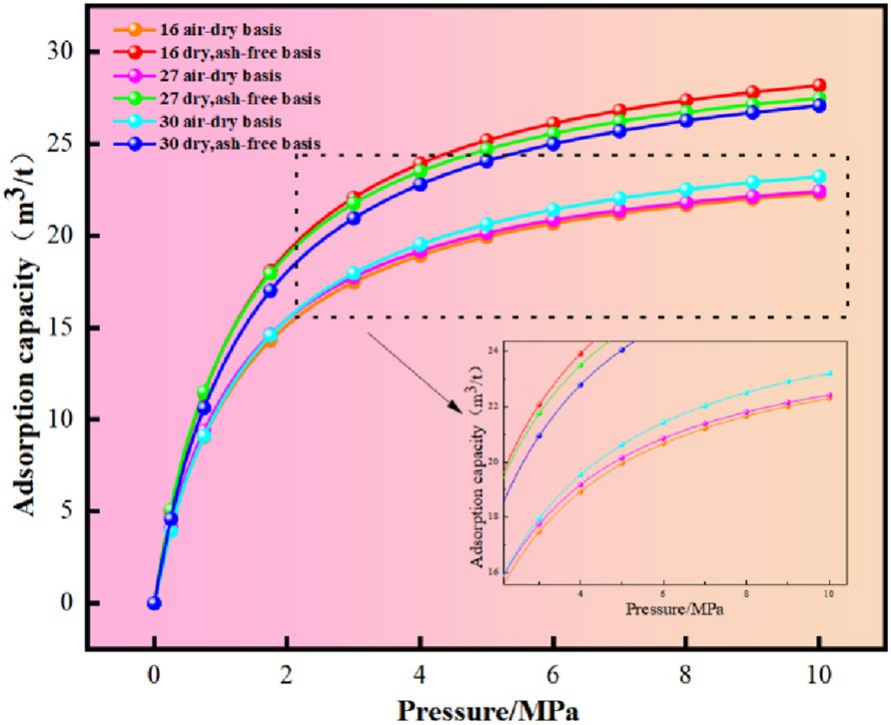
Isothermal adsorption curve of coal seam.

The isothermal adsorption results of each reservoir are shown in Table 3 and Fig. 7. It can be seen from Fig. 7 that under the same pressure conditions, the adsorption capacity of dry ash-free basis is significantly higher than that of air-dried coal, which reflects the influence of ash yield on coal adsorption capacity to a certain extent. Under room temperature and the same pressure, the water loss of different coal seams is No.16 coal seam > No.27 coal seam > No.30 coal seam. For dry ash-free basis, the combustion heating components left by different coal seams are: No.16 coal seam > No.27 coal seam > No.30 coal seam.

**Table 3:** Statistics of isothermal adsorption test results of coal seam.

Coal seam number	Moisture (Mad)/%	Ash (Ad)/%	VL._daf_/m^3^/t	VL._ad_/m^3^/t	PL/Mpa	R_max_/%
16	18.60	0.38	31.98	25.30	1.34	2.55
27	15.29	0.56	30.99	25.28	1.27	3.84
30	11.08	0.75	30.98	26.56	1.44	3.13

Rmax is the maximum vitrinite reflectance; VL.ad is the volume of air drying base; VL.daf is the dry ash-free volume.

The Langmuir volume is an index reflecting the adsorption capacity of coal-generally, the larger its value, the better the adsorption performance^42^. The Langmuir pressure is mainly a parameter that affects the shape of the isothermal adsorption curve, reflecting the pressure when the adsorption capacity reaches half of the Langmuir volume. The larger the index, the easier the desorption of adsorbed gas in the coal seam, and the more favorable the development^43^. Combined with Table 3, according to the actual sampling of coal seam, the air-dried Langmuir volume of No.16 coal seam is 22.57–34.84 m^3^/t, with an average of 25.30 m^3^/t, and the Langmuir pressure is 0.62–1.66 MPa, with an average of 1.34 MPa; the air-dried Kiran volume of No.27 coal seam is 23.95–34.97 m^3^/t, with an average of 25.28 m^3^/t, and the Langmuir pressure is 0.89–2.70 MPa, with an average of 1.27 MPa. The air-dried Kiran volume of No.30 coal seam is 32.63–36.10 m^3^/t, with an average of 26.56 m^3^/t, and the Langmuir pressure is 0.65–2.11 MPa, with an average of 1.44 MPa. The maximum Langmuir volume of No.30 coal seam is 26.56 m^3^/t, which represents its best adsorption performance and high gas storage capacity. The maximum Langmuir pressure of the No.30 coal seam is 1.44 MPa, which means that the easier gas desorption, the more potential gas production and the better recoverability. The development conditions of the No.30 coal seam are more extensive than those of the No.16 and No.27 coal seams.

**Figure 8:**
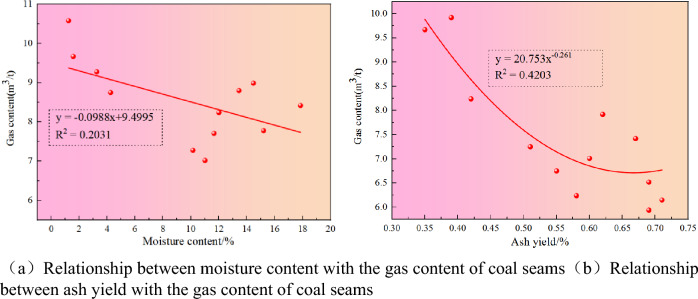
Relationship between moisture content-ash content and gas content.

It can be seen from Fig. 8a that there is a negative correlation between the gas content and moisture content of the coal seam. That is, when the moisture content increases, the gas content of the coal seam decreases. Considering that this phenomenon may be caused by the following two aspects, on the one hand, the overall moisture content of gas content in the study area is relatively small, which may lead to a low correlation between moisture content and gas content of coal seam. On the other hand, combined with Table 3, it can be seen that the maximum range of vitrinite is 2.55–3.84%, indicating that coal has undergone different degrees of metamorphism. The change is low, resulting in low moisture content, which makes the moisture content and coal seam gas content show a common correlation.

The ash in coal is also known as the mineral matter of coal, which is generally not adsorbable. The gas in the coal seam usually refers to the adsorption on the surface of the coal seam, rather than the ash scattered on the surface, which affects the adsorption capacity of the coal. According to the isothermal adsorption curve (Fig. 8), it can be seen that under the same conditions, the adsorption capacity of dry ash-free basis is higher than that of air-dried basis. It can be seen that ash has a certain influence on the adsorption capacity of coal.

### Ground stress and permeability

Ground stress is a crucial factor affecting the permeability of coal reservoirs because it controls the fracture space structure by determining the density, direction, closure, and opening degree of fractures in coal reservoirs^44^. The permeability of coal in this area is extremely sensitive to ground stress, and generally decreases exponentially with the increase of ground stress. The fracture pressure (p_f_), closure pressure (p_c_), pore pressure (p_0_) and permeability of coal seam were obtained by injection pressure drop method. The maximum horizontal principal stress (σ_s3_) can be expressed as^45^:

2 $$ \sigma_{s3} = 3p_{c} - p_{f} + p_{0} + T $$

In the formula, *σ*_*s3*_ is the maximum horizontal principal stress, MPa; *p*_*c*_ is the closing pressure, MPa; *p*_*f*_ is the fracture pressure, MPa; *p*_0_ is coal seam pore pressure (original reservoir pressure), MPa; *T* is the tensile strength of the rock around the borehole, MPa.

The so-called closure pressure is the equilibrium pressure just enough to make the fracture open, which is equivalent to the minimum horizontal principal stress (σ_s1_) perpendicular to the fracture surface, namely:

3 $$ \sigma_{s1} = p_{c} $$

**Figure 9:**
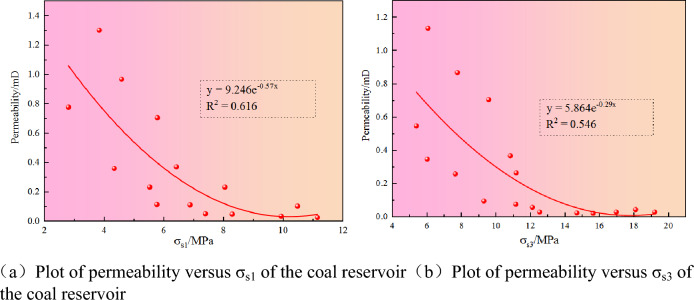
Relationship between horizontal principal stress and permeability.

According to Fig. 9, the horizontal maximum principal stress and the horizontal minimum principal stress exponentially decrease with permeability. Careful observation of Fig. 9 shows that when the horizontal minimum main priority is greater than 10 MPa, the high permeability data disappears; when the horizontal maximum principal stress is greater than 14 MPa, the permeability is extremely low, and its variation range is 0.02–0.08 mD. In summary, ground stress is significant in many influencing factors of coal seam permeability. The relationship between permeability W and in-situ horizontal stress σ_s_ can be expressed as:

4 $$ W_{s1} = 9.246e^{ - 0.57\sigma s1} $$

5 $$ W_{s3} = 5.864e^{ - 0.29\sigma s3} $$

**Figure 10:**
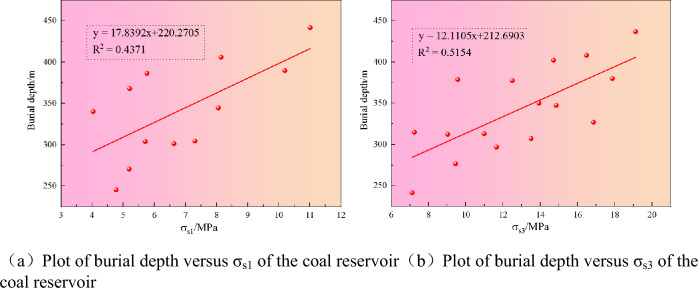
Relationship between horizontal principal stress and buried depth of coal seam.

According to Fig. 10, the horizontal maximum principal stress and the horizontal minimum prominent stress increase with the increase of coal seam burial depth, and R^2^ is 0.4371 and 0.5154, indicating that the ground stress has a specific correlation with the coal seam burial depth. Through further analysis, on the one hand, the ground stress increases with the increase of coal seam depth; on the other hand, the ground stress will reduce the permeability of the coal seam to a certain extent, so the permeability of the coal seam generally decreases with the increase of the buried depth. At the same time, it also shows that the ground stress harms the permeability of the coal seam.

### Measured gas saturation and permeability

The measured saturation is the ratio of the measured gas content to the theoretical gas content corresponding to the measured reservoir pressure projected onto the adsorption isotherm^46^:

6 $$ S_{s} = V_{s} /V $$

where *S*_*s*_ is measured saturation, %; *V*_*s*_ is measured gas content, m^3^/t; *V* is the theoretical gas content corresponding to the measured reservoir pressure projected onto the adsorption isotherm, m^3^/t.

The critical desorption pressure of coalbed methane refers to the pressure at which the gas adsorbed on the surface of coal micropores begins to desorb when the desorption and adsorption reach equilibrium, that is, the pressure corresponding to the measured gas content of coal samples on the isothermal adsorption curve. The calculation formula is as follows^47^:

7 $$ P_{cd} = \frac{{V_{S} P_{L} }}{{V_{L} - V_{S} }} $$

In Eq. (3), *P*_*cd*_ is critical desorption pressure, MPa; *V*_*s*_ is measured gas content, m^3^/t; *P*_*L*_ is Langmuir pressure, MPa; *V*_*L*_ is Langmuir volume, m^3^/t. Combined with formula (6) and formula (7), the gas saturation of coal seam is calculated, and the results are shown in Table 4.

**Table 4:** coal seam gas saturation calculation results table.

Coal seam number	Reservoir pressure/MPa	Theoretical gas content/m^3^/t	Measured gas content/m^3^/t	Degree of saturation/%	Critical desorption pressure/MPa
16	4.80	19.78	13.49	68.2	1.53
27	1.96	15.34	15.19	99.0	1.91
30	5.03	20.65	17.49	84.7	2.78

According to Table 4, the measured gas saturation of the No.16 coal seam is 68.2%, and the critical desorption pressure is distributed at 1.53 MPa. The measured gas saturation of 27 layers is 99.0%, and the necessary desorption pressure is 1.91 MPa. The measured gas saturation of the No.30 coal seam is 84.7%, and the critical desorption pressure is distributed at 2.78 MPa. Due to the low Langmuir pressure, the necessary desorption pressure is low.

The coal reservoir is a dual-porosity medium containing matrix pores and fissure pores (cleats). In the coal seam, micropores occupy most of the pores. Although the proportion of fractures is small, they are the main flow channels of the fluid, providing guidance conditions for the liquid to flow to the bottom of the well.

According to the permeability test results of coalbed methane wells (Table 5), the permeability of No.16 coal seam is 0.04–0.17 mD; No.27 coal seam 0.38–0.85 mD; the No.30 coal seam is 0.02–0.08 mD, indicating that the overall permeability of the coal seam in the region is low.

**Table 5:** Well test results of coalbed methane wells.

Coal seam number	Depth of pressure point/m	Pressure gradient/MPa/100 m	Bursting pressure/MPa	Fracture pressure gradient/MPa/100 m	Closure pressure/MPa	Closed pressure gradient/MPa/100 m	Permeability/mD
16	432.73	1.11	9.84	2.23	9.49	2.15	0.17
27	303.67	0.63	8.50	2.72	8.07	2.58	0.85
30	523.45	0.96	14.18	2.67	12.32	2.32	0.08

**Figure 11:**
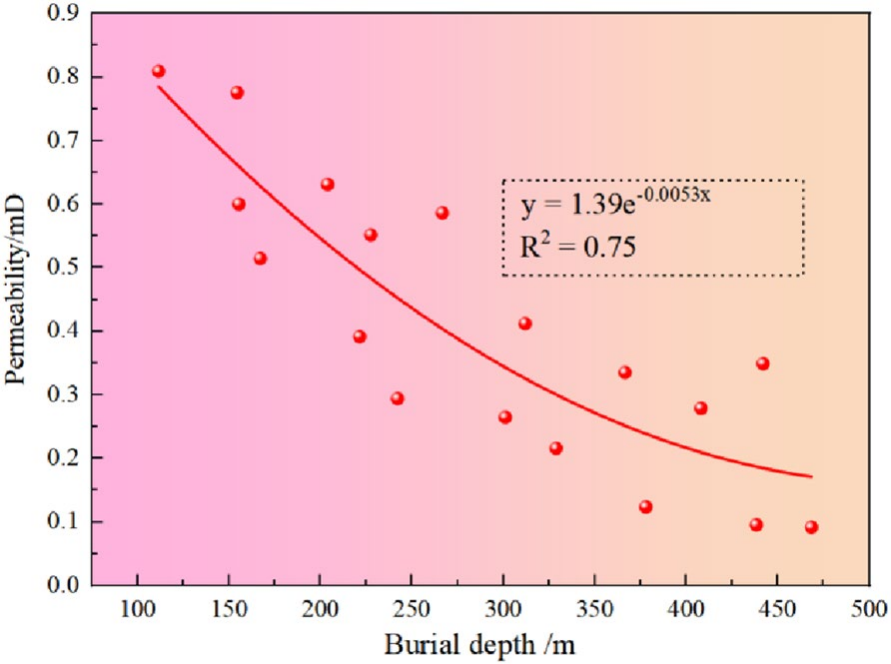
The relationship between permeability and buried depth.

According to the field data, the correlation fitting method is used to fit it. The appropriate results are shown in Fig. 11. It can be seen from Fig. 11 that the buried depth and permeability of the coal seam decrease exponentially (negative correlation), that is, the deeper the buried depth, the smaller the permeability. The reason is that the ground stress increases with the increase of buried depth. However, in most cases, the ground stress harms the permeability, so the permeability decreases with the addition of buried depth.

## Conclusion

1. The thickness of the No.16 coal seam is 0.96–4.05 m, and the average thickness is 1.85 m. The thickness of the No.27 coal seam is in the range of 0.60–3.29 m, and the average thickness is 1.55 m. The thickness of the No.30 coal seam is 0.60–2.71 m, with an average of 1.32 m. The average buried depth of the No.16 coal seam is 301.3 m, the average buried depth of the No.27 coal seam is 366.6 m, and the average buried depth of the No.30 coal seam is 384.2 m. The gas content of the No.16 coal seam forms a gas-rich center in the south of the W1 mine, and the gas content in the syncline core is higher. The gas content of the No.27 coal seam is higher in the heart of the syncline, which forms a gas-rich center in the south, middle, and north of the W1 mine. The gas content of the No.30 coal seam constitutes a gas-rich center in the south of the W1 mine.
2. The buried depth and gas content of the coal seam in the W1 thriving area and W2 thriving well show a strong positive correlation, R^2^ = 0.6142; there is a negative correlation between the gas content and moisture content of the coal seam, which may be caused by the overall moisture content of the gas content in the study area and the different degrees of metamorphism of the coal. Under the same conditions, the adsorption capacity of dry ash-free basis is higher than that of air dry basis, which shows that ash has a particular influence on the adsorption capacity of coal.
3. The horizontal maximum principal stress and the horizontal minimum principal stress exponentially decrease with the permeability. When the horizontal minimum principal stress is greater than 10 MPa, the data of high permeability disappears; The permeability of the No.16 coal seam is 0.04–0.17 mD; No.27 coal seam 0.38–0.85 mD; The No.30 coal seam is 0.02–0.08 mD, indicating that the overall permeability of the coal seam in the region is low. The buried depth and permeability of coal seam decrease exponentially. The reason is that the ground stress increases with the increase of concealed depth, and the ground stress generally harms the permeability, so the permeability decreases with the growth of buried depth.

## Data Availability

All data used during this research are available from the corresponding author by reasonable request.
